# Effects of *Chinese yam* polysaccharides on the immune function and serum biochemical indexes of broilers

**DOI:** 10.3389/fvets.2022.1013888

**Published:** 2022-09-06

**Authors:** Jiahua Deng, Jinzhou Zhang, Yadi Chang, Suli Wang, Mingyan Shi, Zhiguo Miao

**Affiliations:** ^1^College of Animal Science and Veterinary Medicine, Henan Institute of Science and Technology, Xinxiang, China; ^2^Life Science College, Luoyang Normal University, Luoyang, China

**Keywords:** *Chinese yam* polysaccharides, broilers, immune organ index, cytokine, serum

## Abstract

The purpose of this experiment was to investigate the effects of *Chinese yam* polysaccharides (CYP) in diets on the immune function of broilers. A total of 360 (1-day-old, sex balance) healthy growing broilers with similar body weight (39.54 ± 0.51 g) were randomly divided into control (0.00 g/kg), CYP I (0.25 g/kg), CYP II (0.50 g/kg), and CYP III (1.00 g/kg) groups. Each group contains 3 replicates with 30 broilers in each replicate, and the feeding trial lasted 48 d. The results showed that compared with the control group, the CYP II group had higher thymus index, serum IgA, complement C3, C4, IGF-I, T_3_, T_4_, INS, GH, IL-2, IL-4, IL-6, and TNF-α levels (*P* < 0.05) at 28, 48 d, respectively. In addition, the spleen index, serum IgM and IgG concentrations in CYP II group were higher than those in the control group at 28 d (*P* < 0.05). Results indicated that 0.50 g/kg CYP supplementation improved the immune function of broilers, and the CYP has a potential biological function as a green additive in broilers.

## Introduction

In intensive breeding and production, improving the nutrient utilization rate and immunity are the key to ensure efficiency and health in the livestock and poultry industry (1, 2). However, antibiotic abuse in actual production has caused a tremendous negative influence on the livestock and poultry industry, such as producing antibiotic residues, inducing bacterial resistance and reducing animal immune function. In 2020, the Chinese government banned the addition of antibiotics to animal feed. Therefore, the research and development of safe, efficient and green antibiotic substitutes for livestock and poultry are necessary. Plant-active polysaccharides are macromolecules with a variety of biological activities that can improve the growth performance and immune function in poultry. At present, these substances have become the research hotspot of antibiotic substitutes for livestock and poultry (3, 4).

*Chinese yam* (CY) is the dried rhizome of *Dioscoreae* plant that grows all over China and is a traditional Chinese herbal medicine with high edible value (5). The polysaccharide isolated from CY has low toxicity, high biological activity and biological characteristics of promoting growth, regulating immunity and the gastrointestinal tract and reducing blood sugar (6–8). CYP can induce a specific immune response in animals, increase the number of immune cells, enhance the immune activity and phagocytosis of immune cells and stimulate the production of immunoglobulin and cytokines, thus regulating the immune function *in vivo* and *in vitro* and improving the cellular and humoral immune function of the body (9). CYP also plays essential roles in animals, such as improving mice insulin sensitivity; playing an anti-hyperlipidaemic role (10); promoting the proliferation of spleen lymphocytes; significantly increasing the levels of cytokines, and serum immunoglobulins in mice, and decreasing the content of total cholesterol (TC) and triglyceride (TG) in rat serum (11, 12). Dietary CYP supplementation also significantly increased the contents of tumor necrosis factor α (TNF-α) and IL-2 in the serum of weaned rats, suggesting that CYP can improve the immune response of rats (7).

CYP can improve immune function and serum biochemical indicators in mice, rats and pigs; however, its effect on immune organ index, serum immunoglobulins, complements and cytokines in broilers is unknown. In this study, the hypothesis is that different amounts of CYP could change the immune organ index, serum immunoglobulins, complements and cytokines of broilers, thereby improving their immune function and serum biochemical indexes. Therefore, an experiment was conducted to examine the effect of different amounts of dietary CYP addition on the immune function and serum biochemical indexes of broilers and to provide a theoretical basis for rational dietary CYP addition in broiler production.

## Materials and methods

### Experimental diets and animal management

A total of 360 (1 day old, gender-balanced) healthy growing broilers with similar body weight (39.54 ± 0.51 g) were randomly divided into four groups (Control, CYP I, CYP II and CYP III) individually consisting of three replicates with 30 broilers each. The control group was fed a basal diet without CYP, and the CYP I, CYP II and CYP III groups were fed a basal diet added with 0.25, 0.50 and 1.00 g/kg CYP, respectively. Feeding trials were divided into an initial phase (1–28 days) and an end phase (29–48 days). The basal diet was formulated to meet or exceed the NRC (1994) nutrient requirements for broilers. The composition and nutrient levels of the basal diet are shown in Table 1. During the whole trial, all broilers had *ad libitum* access to equally complete feed and water using nipple drinkers. The data on feed intake were a part of another article that is being prepared for publication elsewhere. In simple terms, the average daily feed intake (g/d) of the following: 1–28 d (23.50 ± 0.75 control group, 27.36 ± 0.23 CYP I group, 28.88 ± 0.03 CYP II group and 26.06 ± 0.01 CYP III group), 29-48 d (89.16 ± 1.38 control group, 97.72 ± 0.02 CYP I group, 98.78 ± 0.09 CYP II group and 97.19 ± 0.03 CYP III group),1-48 d (56.33 ± 0.50 control group, 62.54 ± 0.14 CYP I group, 63.83 ± 0.02 CYP II group and 61.62 ± 0.04 CYP III group). The broilers were reared in appropriate confinement cage with controlled air temperature. The light was continuously turned on for 24 hours, and the temperature was held at 32°C for the first 3 days and then gradually cooled until 26 °C from days 4–21. The CYP in the study was purchased from Shanxi Hana Biotechnology Co., Ltd. (Shanxi, China) and had a carbohydrate level of sed from(CYP content a caramong them, monosaccharide types including glucose 99.48%, galactose 0.52%).

**Table 1:** Ingredient and nutrient levels of the basal diet in each feeding phase for broilers.

**Items**	**Composition (%)**	
	**Day 28**	**Day 48**
**Ingredients (%)**
Corn	60.00	63.50
Soybean meal	32.00	29.00
Wheat bran	1.00	-
Soybean oil	1.00	2.00
Fish meal	2.00	1.60
CaHPO_4_	1.30	1.30
Limestone	1.40	1.30
NaCl	0.30	0.30
Premix^a^	1.00	1.00
Total	100.00	100.00
**Nutrient levels (%)**
Metabolic energy, (MJ/kg)^b^	12.13	12.55
Crude protein	21.00	20.00
Calcium	1.00	0.90
Total P	0.65	0.60
Available P	0.45	0.35
Lysine	0.50	0.38
Methionine	1.10	1.00

^a^Premix supplied per kg: VA 3000 IU,VD_3_ 500 IU, VE 10 IU, VK_3_ 0.5 mg,VB_6_ 3.5 mg, VB_1_ 3.8 mg, D-pantothenic acid 10 mg, folie acid 0.5 mg, biotin 0.15 mg, Fe 80 mg, Cu 8 mg, Zn 75 mg, Mn 60 mg, Se 0.15 mg.

^b^Metabolic energy was calculated by value and others were measured values.

### Blood sample collection

At 28 and 48 days, 24 broilers (six broilers of similar body weight in each group, two broilers per replicate, gender balance) of similar weight were randomly selected to collect blood samples. After weighing, the blood was collected from the wings' veins of broilers, placed in a centrifuge tube and allowed to coagulate at 4 °C. After centrifugation (3,000 g, 10 min, at 4°C), the serum was collected into sterile tubes and stored at −80 °C until analysis.

### Determination of immune organ index

At 28 and 48 days, the experimental broilers were slaughtered. The thymus, bursa of Fabricius (BF) and spleen were dissected from each experimental chicken; the surface blood of the immune organ was wiped dry with clean absorbent paper and the connective tissues surrounding the immune organ were dissected and weighed. The formula for immune organ index was adopted from Chi (13) as follows:


$$Immune\;organ\;index=\frac{Immune\;organ\;weight\;\left(g\right)}{Live\;weight\;of\;broiler\;\left(kg\right)}\;$$

### Assay of serum immunoglobulins and complements C3 and C4

At 28 and 48 days, the serum concentrations of IgM, IgA and IgG and complements C3 and C4 in experimental broilers were measured using respectively chicken IgM, IgA, IgG ELISA Kits, chicken C3 and C4 ELISA Kits purchased from Nanjing Jiancheng Institute of Biological Engineering (Nanjing, China).

### Determination of serum biochemical indicators

At days 28 and 48, the serum concentrations of insulin-like growth factor I (IGF-I), insulin (INS), growth hormone (GH), triiodothyronine (T3) and thyroxine (T4) in chicken were measured using respectively chicken IGF-I, INS, GH, T3, T4 ELISA Kits (Beijing Northern Institute of Biotechnology, Beijing, China).

### Assay of serum cytokines

At days 28 and 48, the serum concentrations of TNF-α and serum cytokine interleukin (IL)-2, IL-4 and IL-6 were determined using respectively chicken ELISA kits (Nanjing Jiancheng Institute of Biological Engineering Nanjing, China) in accordance with the manufacturer's instructions.

### Statistical analysis

Statistical analysis of variance (ANOVA) was performed using the one-way ANOVA procedure of SPSS 26.0 for Windows (IBM Corp., Chicago, IL, USA). Significant differences among all treatments were measured at *P* < 0.05 by Duncan's multiple range tests. All data were presented as mean ± SEM (standard error of the means).

## Results

### Immune organ index

As shown in Table 2, the immune organ index of the thymus in the CYP II group was significantly higher than that in the control group at days 28 and 48. Meanwhile, the immune organ index of the spleen in the CYP II group was significantly higher than in the control group at day 28. The immune organ index of BF in the CYP II group was significantly higher than that in the CYP I and CYP III groups at day 48. Meanwhile, no significant difference among the four groups was found for the immune organ index of BF at day 28 and of spleen at day 48.

**Table 2:** Effects of CYP on the immune organ index in broilers (g/kg).

	**CYP level (g/kg)** ^**1**^			
**Item**	**Control**	**CYP I**	**CYP II**	**CYP III**	**SEM**	***P*-value**
**Day 28**
Thymus	3.42^b^	5.02^a^	5.59^a^	4.70^a^	0.427	0.006
BF	0.83	0.97	1.00	0.96	0.960	0.395
Spleen	1.79^c^	1.85^bc^	2.21^a^	2.12^ab^	0.136	0.039
**Day 48**
Thymus	2.82^b^	3.53^ab^	4.29^a^	3.06^b^	0.351	0.014
BF	0.66^a^	0.55^b^	0.60^ab^	0.43^c^	0.435	0.004
Spleen	1.86	2.31	2.38	2.24	0.394	0.582

In the same line, values with different lowercase superscripts indicate significant differences, and values with the same lowercase superscripts indicate no significant differences (P < 0.05). n = 6. BF, bursa of fabricius.

^1^CYP I, CYP II, CYP III represented the data of broilers fed with 0.25 g, 0.50 g, and 1.00 g CYP per kilogram of diet, respectively.

### Serum immunoglobulins and complements C3 and C4

The effects of dietary CYP on the concentrations of serum immunoglobulins and complements C3 and C4 in broilers are shown in Table 3. The serum concentrations of IgA and complements C3 and C4 in the CYP II group were significantly higher than those in the control group at days 28 and 48 (*P* < 0.05). In addition, the serum IgG concentrations in the CYP II group were higher than those in the control, CYP I and CYP III groups at day 28 (*P* < 0.05). Meanwhile, no differences in serum IgM and IgG concentrations were found among all the groups at day 48 (*P* > 0.05).

**Table 3:** Effects of CYP on the concentrations of serum immunoglobulins and complements C3 and C4 in broilers.

	**CYP level (g/kg)** ^**1**^			
**Item**	**Control**	**CYP I**	**CYP II**	**CYP III**	**SEM**	***P*-value**
**Day 28**
IgM (ng/mL)	5866.64^b^	6056.54^b^	6369.87^a^	6365.12^a^	82.707	0.001
IgA (ng/mL)	8588.37^c^	9049.87^ab^	9331.47^a^	8862.14^bc^	152.481	0.007
IgG (ng/mL)	85.98^c^	92.09^b^	96.21^a^	89.36^b^	1.374	0.001
C3 (μg/mL)	713.88^c^	867.57^a^	874.65^a^	824.37^b^	15.540	0.001
C4 (μg/mL)	458.04^c^	478.37^b^	560.58^a^	556.06^a^	8.206	0.001
**Day 48**
IgM (ng/mL)	5097.55	5092.81	5197.25	5140.28	72.155	0.481
IgA (ng/mL)	7915.66^b^	8158.15^ab^	8447.57^a^	8087.75^b^	146.233	0.038
IgG (ng/mL)	77.95	78.02	79.05	78.09	1.367	0.832
C3 (μg/mL)	747.88^c^	845.62^ab^	859.78^a^	810.21^b^	15.612	0.001
C4 (μg/mL)	519.02^b^	511.79^b^	570.52^a^	561.48^a^	7.564	0.001

In the same line, values with different lowercase superscripts indicate significant differences, and values with the same lowercase superscripts indicate no significant differences (P < 0.05). n = 6.

^1^CYP I, CYP II, CYP III represented the data of broilers fed with 0.25 g, 0.50 g, and 1.00 g CYP per kilogram of diet, respectively.

### Serum biochemical indicators

The effects of dietary CYP on serum biochemical indicators in broilers are shown in Table 4. The serum concentrations of IGF-I, INS, GH, T_3_ and T_4_ in the CYP II group were significantly higher than those in the control group at days 28 and 48 (*P* < 0.05). In addition, the serum concentrations of GH, T_3_ and T_4_ in the CYP II group at day 28 and the serum concentrations of IGF-I and T_3_ at day 48 were significantly higher than those in the control, CYP I and CYP III groups (*P* < 0.05).

**Table 4:** Effects of CYP on the serum biochemical indicators in broilers.

	**CYP level (g/kg)** ^**1**^			
**Item**	**Control**	**CYP I**	**CYP II**	**CYP III**	**SEM**	***P*-value**
**Day 28**
IGF-I (μg/L)	36.11^b^	38.62^a^	38.99^a^	35.91^b^	0.655	0.002
INS (mU/L)	17.00^b^	19.02^a^	19.42^a^	17.03^b^	0.310	0.001
GH (μg/L)	18.24^d^	19.59^c^	21.51^a^	20.21^b^	0.243	0.001
T_3_ (pmol/L)	314.21^c^	340.65^b^	368.02^a^	338.20^b^	6.176	0.001
T_4_ (pmol/L)	925.12^b^	964.36^b^	1151.82^a^	925.12^b^	24.939	0.001
**Day 48**
IGF-I (μg/L)	34.80^c^	36.51^b^	41.09^a^	37.71^b^	0.487	0.001
INS (mU/L)	16.77^c^	17.56^b^	20.18^a^	19.76^a^	0.225	0.001
GH (μg/L)	20.77^b^	22.48^a^	23.35^a^	22.96^a^	0.348	0.001
T_3_ (pmol/L)	296.08^d^	308.68^c^	346.50^a^	331.13^b^	5.198	0.001
T_4_ (pmol/L)	925.12^c^	1125.67^a^	1181.25^a^	1030.84^b^	17.676	0.001

In the same line, values with different lowercase superscripts indicate significant differences, and values with the same lowercase superscripts indicate no significant differences (P < 0.05). n = 6.

^1^CYP I, CYP II, CYP III represented the data of broilers fed with 0.25 g, 0.50 g, and 1.00 g CYP per kilogram of diet, respectively.

### Serum cytokines

As shown in Table 5, the serum concentrations of TNF-α, IL-2, IL-4 and IL-6 in the CYP II group were significantly higher than those in the control group at days 28 and 48 (*P* < 0.05). In addition, the serum concentrations of IL-2 and IL-6 in the CYP II group were significantly higher than those in the CYP I and CYP III groups at days 28 and 48 (*P* < 0.05).

**Table 5:** Effects of CYP on the serum levels of cytokine TNF-a and IL-2, IL-4 and IL-6 levels in broilers.

	**CYP level (g/kg)** ^**1**^			
**Item**	**Control**	**CYP I**	**CYP II**	**CYP III**	**SEM**	***P*-value**
**Day 28**
TNF-α (ng/L)	60.64^b^	61.15^b^	65.77^a^	63.89^a^	1.157	0.007
IL-2 (ng/L)	143.34^d^	152.06^c^	170.44^a^	161.40^b^	2.472	0.001
IL-4 (ng/L)	168.19^b^	184.45^b^	204.74^a^	202.81^a^	12.733	0.046
IL-6 (ng/L)	40.78^d^	44.09^c^	51.62^a^	49.31^b^	0.573	0.001
**Day 48**
TNF-α (ng/L)	64.79^b^	68.27^a^	70.04^a^	68.96^a^	1.105	0.007
IL-2 (ng/L)	179.47^c^	184.38^c^	214.17^a^	204.19^b^	2.347	0.001
IL-4 (ng/L)	182.04^c^	195.40^b^	202.32^a^	181.88^c^	1.821	0.001
IL-6 (ng/L)	42.65^d^	45.47^c^	51.58^a^	49.00^b^	0.625	0.001

In the same line, values with different lowercase superscripts indicate significant differences, and values with the same lowercase superscripts indicate no significant differences (P < 0.05). n = 6.

^1^CYP I, CYP II, CYP III represented the data of broilers fed with 0.25 g, 0.50 g, and 1.00 g CYP per kilogram of diet, respectively.

## Discussion

The thymus, BF and spleen are the most critical immune organs of chickens. The development of immune organs is closely related to the immune organ index, which is usually used to reflect the immune function and health status of animals (14). In this experiment, 0.50 g/kg CYP supplementation significantly increased the immune organ index of broilers. As a result, the immune level of broilers was regulated. Numerous evidences demonstrated that plant-active polysaccharides can increase the index and enhance the status of animal immune organs. Song et al. (15) showed that 5 mg/kg supplementation of medicinal mushroom *Antrodia camphorata* polysaccharides significantly increased the immune organ index of spleen and BF in aseptic chickens compared with those in the control group after 14 days. Liang et al. (16) reported that 200 mg/ml supplementation of Taishan *Robinia pseudoacacia* polysaccharides can significantly increase the index of the spleen, BF and thymus and promote the growth and development of immune organs in chickens. Nan et al. (17) found that 50, 100 and 200 mg/kg supplementation of *Lycium barbarum* polysaccharides (LBP) can increase the immune organ index of the thymus and spleen in mice and improve the animal's immune function. Zhao et al. (18) also found that 0.6 and 1.2 g/kg mulberry leaf polysaccharides (MLPs) additives could significantly increase the thymus and spleen indexes and help enhance the immune performance of piglets. Wang et al. (19) confirmed that 200 mg/kg dietary supplementation of *Camellia oleifera* cake polysaccharides increased the spleen weight and index of yellow feather broilers after 42 days compared with those in the control group, indicating that oil tea polysaccharides have positive effects on the immunity of broilers. Li et al. (20) observed that 50 mg/mL supplementation of Taishan *Pinus massoniana* pollen polysaccharide could significantly increase the immune organ index of the thymus, BF and spleen, thereby improving the immunity efficacy of immunosuppressed chickens. Combined with the above findings on plant-active polysaccharides, the present results showed that CYP can increase the relative weight and index of immune organs by promoting their growth and development and further improving their immune performance.

As important immunologically active substances, immunoglobulins (IgM, IgA, and IgG) and complements (C3, C4) are the main components of humoral immunity, which can enhance the body's immune response. The experiment results showed that adding 0.50 g/kg CYP to the diet can increase the serum levels of IgM, IgA, IgG, C3 and C4 in broilers. Previous study reported that polysaccharides are natural active components in plants and one of the best options for future immune enhancers in animals. Long et al. (21) observed that 2000 mg/kg supplementation of LBP can significantly increase the serum IgM and IgA concentrations of chickens. This discovery is beneficial in enhancing the immune function of broilers. Zhao et al. (18) also found that adding 0.6 g/kg MLPs to the diet significantly increases serum IgG levels in weaned piglets, thus effectively improving their immune capacity. Liu et al. (22) confirmed that 0.2 g/L supplementation of *Hericium erinaceus* polysaccharides can significantly increase the serum levels of IgM, IgA, IgG and complements C3 and C4 in Muscovy duck, thus improving its immune function. Wu (4) showed that 1 g/kg supplementation of *Astragalus membranaceus* polysaccharides (AMP) increased the serum levels of IgM, IgA and IgG in broilers, indicating that AMP can be used as an immunostimulator to improve the immune function of broilers. Chen et al. (23) reported that adding 4000 mg/kg of LBP to the diet significantly increased the serum IgM and IgG levels of weaned piglets compared with those of the control group, indicating that LBP can enhance the immune status of weaned piglets. All of these results suggest that the increase of serum immunoglobulins and complements may be related to the kind of polysaccharides, type of animals, addition amount of polysaccharide, feeding period and feeding method.

This experimental study found that adding 0.50 g/kg CYP to the diet can increase the serum concentrations of IGF-I, INS, GH, T_3_ and T_4_ in broilers. These results indicated that CYP might improve the growth and metabolism of broilers by regulating their serum hormone levels. A previous study found that a daily injection of 100 mg/kg *Schisandra challenges* acidic polysaccharides for 8 weeks effectively increased the fasting INS concentrations in rats with type 2 diabetes (24). Wu et al. (25) confirmed that 0.2% supplementation of *Radix rehmanniae preparata* polysaccharides could increase the serum GH level in alpaca, thus improving its growth performance. Zeng et al. (26) also found that the 50 mL injection of APS for 21 consecutive days had a positive effect on increasing the serum concentrations of T_3_ and T_4_ in heat-stressed cows. These results showed that different plant-active polysaccharides could improve the serum hormone levels of animals to varying degrees and were consistent with the present experimental results. However, how the yam polysaccharide regulates the serum hormone level of broilers and its potential mechanism need to be further studied.

When the body is invaded by pathogens, it will initiate an immune response and protect itself by releasing a series of cytokines (11). TNF-α and IL-6 cytokines play eliminate inflammation, and TNF-α can influence other cells to produce additional inflammatory cytokines that will participate in the body's immune response (27, 28). IL-2 and IL-4 are essential regulators of cellular immune function and are secreted by the Th1 and Th2 cell subgroups, respectively (29, 30). Increasing evidences indicated that plant-active polysaccharides could regulate the levels of cytokines, such as TNF-α, IL-2, IL-4 and IL-6. Long et al. (21) found that supplementing 2000 mg/kg LBP in the diet can significantly increase the levels of TNF-α and IL-4 compared with those in the control group, thus confirming that LBP can enhance the immune activity of chickens. Park et al. (31) observed that the supplementation of *Platycodon grandiflorum* polysaccharides could increase the ability of mouse dendritic cells to secrete TNF-α, IL-2 and IL-6. Zhang et al. (32) reported that 50 and 100 mg/kg APS supplementation can promote chicken's lymphocyte proliferation and enhance the immune response of TNF-α and IL-2. Li et al. (20) observed that 50 mg/mL supplementation of Taishan *Pinus massoniana* pollen polysaccharides can significantly increase the IL-2 level of immunosuppressed chickens, proving that these polysaccharides could promote the immune regulation ability of cytokines. Mirzaie et al. (33) showed that the supplementation of *Chlorella Vulgaris* polysaccharides could induce peripheral blood mononuclear cells and promote IL-2 expression in broilers. In the present study, 0.50 g/kg CYP supplementation increased the serum concentrations of TNF-α, IL-2, IL-4 and IL-6. Our results were similar to the above reports and indicated that CYP can improve the immune activity and promote the immune regulation ability of cytokines.

In our research, the concentration of immune parameters, serum IgM, IgA, IgG, C3, C4, IL-2, IL-4, IL-6 and TNF-α, were decreased when the concentration of CYP was increased to 1.00 g/kg, Similar results have been reported in previous studies (4, 21). The reason may be that the high concentration of plant-active polysaccharides affects the emulsification, thus reducing the immune parameters of these experiment. However, the mechanism by which high concentrations of plant-active polysaccharides lead to decreased immune parameters requires further study.

## Conclusion

The dietary supplementation of 0.50 g/kg CYP improved the immune organ index and the serum immunoglobulin, complement and cytokine levels of broilers. However, with the dietary supplementation of 1.00 g/kg CYP, the promoting effect of CYP tended to decrease. These data suggested that the dietary supplementation of 0.50 g/kg CYP can effectively enhance the immune function of broilers.

## Data availability statement

The original contributions presented in the study are included in the article/supplementary files, further inquiries can be directed to the corresponding author.

## Ethics statement

The animal study was reviewed and approved by Animal Protection and Utilization Committee of Henan Institute of Science and Technology.

## Author contributions

JD: investigation, writing-original manuscript, and writing–review and editing. JZ, YC, and SW: data curation and formal analysis. MS: conceptualization, methodology, and supervision. ZM: project administration and funding acquisition. All authors contributed to the article and approved the submitted version.

## Funding

This research was supported by the Program for Innovative Research Team (in Science and Technology) in University of Henan Province (22IRTSTHN026) and Provincial Key Technology Research and Development Program of Henan (212102110180, 212102110169, and 202102110092).

## Conflict of interest

The authors declare that the research was conducted in the absence of any commercial or financial relationships that could be construed as a potential conflict of interest.

## Publisher's note

All claims expressed in this article are solely those of the authors and do not necessarily represent those of their affiliated organizations, or those of the publisher, the editors and the reviewers. Any product that may be evaluated in this article, or claim that may be made by its manufacturer, is not guaranteed or endorsed by the publisher.
